# Computational Study of the pH-Dependent Ionic Environment around β-Lactoglobulin

**DOI:** 10.1021/acs.jpcb.2c03797

**Published:** 2022-11-02

**Authors:** Lucie da Rocha, António M. Baptista, Sara R. R. Campos

**Affiliations:** Instituto de Tecnologia Química e Biológica António Xavier, Universidade Nova de Lisboa, Avenida da República, 2780-157 Oeiras, Portugal

## Abstract

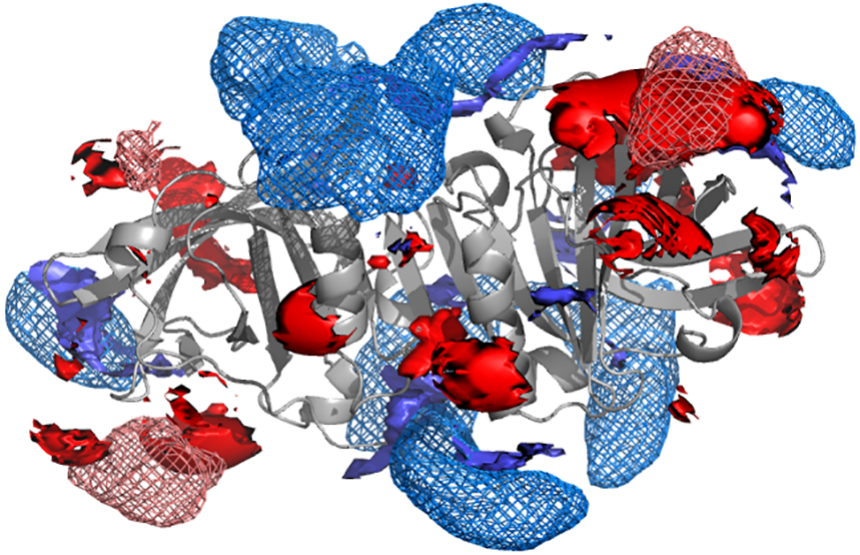

Ions are involved in multiple biological processes and may exist bound to biomolecules or may be associated with their surface. Although the presence of ions in nucleic acids has traditionally gained more interest, ion–protein interactions, often with a marked dependency on pH, are beginning to gather attention. Here we present a detailed analysis on the binding and distribution of ions around β-lactoglobulin using a constant-pH MD (CpHMD) method, at a pH range 3–8, and compare it with the more traditional Poisson–Boltzmann (PB) model and the existing experimental data. Most analyses used ion concentration maps built around the protein, obtained from either the CpHMD simulations or PB calculations. The requirements of approximate charge neutrality and ionic strength equal to bulk, imposed on the MD box, imply that the absolute value of the ion excess should be half the protein charge, which is in agreement with experimental observation on other proteins (Proc. Natl. Acad. Sci. U.S.A.2021, 118, e201587911833372141) and lends support to this protocol. In addition, the protein total charge (including territorially bound ions) estimated with MD is in excellent agreement with electrophoretic measurements. Overall, the CpHMD simulations show good agreement with the nonlinear form of the PB (NLPB) model but not with its linear form, which involves a theoretical inconsistency in the calculation of the concentration maps. In several analyses, the observed pH-dependent trends for the counterions and co-ions are those generally expected, and the ion concentration maps correctly converge to the bulk ionic strength as one moves away from the protein. Despite the overall similarity, the CpHMD and NLPB approaches show some discrepancies when analyzed in more detail, which may be related to an apparent overestimation of counterion excess and underestimation of co-ion exclusion by the NLPB model, particularly at short distances from the protein.

## 1 Introduction

Solution ions are an ubiquitous presence in biological processes, modulating biomolecular function in a multitude of ways, from their central role in ionic transport and storage to the direct control of biomolecular mechanisms by specific structural ions and to the more subtle but equally crucial effect of loosely associated ions on biomolecular stability, association, and solubility. The interaction of ions with nucleic acids has traditionally arisen more interest due to the marked ionic condensation induced by the high charge density of these molecules,^1^ but ion–protein interactions are receiving increasing attention.^2,3^

The binding of ions to biomolecules can be classified as either site-specific or territorial.^4^ Site-bound ions interact directly with a specific location on (or beneath) the surface of the biomolecule, usually without intervening waters, being often visible in crystallographic or NMR structures. Territorially bound ions are weakly associated with regions of high charge density on the surface of the biomolecule, being more elusive to experimental detection. Their distribution around biomolecules can be experimentally characterized with anomalous small-angle X-ray scattering,^5^ and they can be quantified by electrophoretic methods^2^ or by combining buffer equilibration with atomic emission spectroscopy,^6^ inductively coupled plasma mass spectroscopy^7^ or NMR,^8,9^ with the latter allowing also for direct determination of electrostatic potentials.^10^

Among theoretical approaches, Poisson–Boltzmann (PB) models are a traditional route to study ionic effects on nucleic acids and proteins.^11,12^ However, besides treating the solvent as a homogeneous dielectric, PB models neglect ion–ion correlations and ion size effects, showing some limitations when dealing with the high charge density of nucleic acids.^1,13,14^ Several PB studies have focused on the binding of ions to proteins, including the use of electrostatic potential-based clustering,^15,16^ possibly supplemented with ionic Born desolvation penalties,^17^ and the addition of explicit grand canonical Monte Carlo sampling of ions on top of the PB model itself.^18^

Molecular dynamics (MD) simulations using molecular mechanics force fields provide a general alternative to study the interaction of ions with biomolecules. In the case of ion binding to nucleic acids, MD tends to agree with experimental data significantly better than PB.^13,19^ In the case of ion–protein interactions, MD has been playing a major role in clarifying the molecular-level origins and limitations of the Hofmeister series.^20−22^

In the present article, the binding and distribution of ions around proteins are investigated using the molecular dynamics (MD) simulations of β-lactoglobulin (BLG) that we have recently conducted for both the monomeric and dimeric forms using a constant-pH MD (CpHMD) method, from pH 3 to 8.^23^ This pH range covers a wide variation of the protein charge due to its protonatable groups (from around +15 to −10 for the monomer, and twice those values for the dimer), which is actually fluctuating during the simulations, as would be the case in solution. So, although no experimental studies specifically directed at the binding and distribution of ions around BLG seem to have been published, these simulations provide an interesting case study to analyze such features in detail and to compare our results with some of the general trends found in other studies. In particular, we make a detailed comparison between the results from our MD simulations and those obtained with a Poisson–Boltzmann model, as well as with available experimental data for other proteins. In addition, there is not a standard way to include ions in CpHMD simulations, with protocols ranging from noninclusion^24^ to neutralization using charge-leveling.^25,26^ Thus, we also investigate how sound is our protocol for ion addition, which uses approximate charge neutrality and ionic strength equal to that in the bulk.

## 2 Theory and Methods

### 2.1 Constant-pH MD Simulations

The constant-pH MD simulations from ref (23) were used in this work. These simulations were performed using the stochastic titration method developed by Baptista and co-workers^27,28^ that consists in performing molecular mechanics/molecular dynamics (MM/MD) simulations using protonation states periodically updated to reflect pH according to Poisson–Boltzmann (PB) and Monte Carlo (MC) calculations. The simulated system consisted in the monomer or the dimer of bovine β-lactoglobulin in SPC water.^29^ The ions Na^+^ and Cl^–^ were also present in the simulation box at concentrations that would keep the system approximately neutral and make ionic strength 0.1 M. The GROMOS 54A7 force field^30^ was used. The simulations were performed at pH values 3, 4, 5, 6, 7, and 8, and the sites allowed to titrate in that pH range were Asp, Glu, His, Nter, and Cter. For the monomer, 4 × 100 ns simulations were performed, and for the dimer, 8 × 100 ns simulations were done; the first 30 ns were discarded prior to analysis. The GROMACS package (version 4.0.7)^31^ was used for the MM/MD simulations, the MEAD package (version 2.2.9)^32^ was used for the linear PB calculations, and the PETIT program (version 1.6)^33−35^ was used for the MC simulations. For further details on CpHMD simulations, see Table S1 in Supporting Information.

### 2.2 Ion Concentration Maps

The Na^+^ and Cl^–^ concentrations at each point **r** around BLG, *c*_+_(**r**) and *c*_–_(**r**), were calculated as discrete grid maps derived in two alternative ways: from the MD simulations and from PB calculations. These grid maps were used for several analyses, including the determination of iso-concentration contours and the calculation of the total number of ions *N*_*i*_ found in a given region , given by
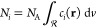
1where *N*_A_ is the Avogadro number.

The MD-derived concentration maps were computed from the ionic densities in the MD box. The probability density of each ion species was calculated with the program LandscapeTools^35,36^ in a grid of mesh size 1 Å using a triangular kernel estimator^36,37^ of bandwidth 2 Å. This used the positions of the Na^+^ and Cl^–^ ions when the protein was fitted to a central structure^36^ and centered in the dodecahedral box containing the system. Since the MD simulations were conducted in a rhombic dodecahedral box with periodic boundary conditions, while the kernel density estimation was done in a nonperiodic rectangular grid, bins near the box boundary must be avoided in most analyses. In practice, this was done by limiting the analyses to bins within a given maximum distance from the closest protein atom, namely around 15 Å for the monomer and 25 Å for the dimer.

The PB-derived concentration maps were estimated from the electrostatic potential ϕ(**r**) using the Debye–Hückel approximation^38,39^
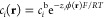
2where  is the concentration of ionic species *i* in the bulk, *z*_*i*_ is its charge (in protonic units), *F* is the Faraday constant, *R* is the gas constant, and *T* is the absolute temperature. The electrostatic potential was calculated using the PB equation, and both the linear (LPB) and the nonlinear (NLPB) forms were tested. These concentrations were calculated for a representative state that had the average charges sampled by the CpHMD simulations and the conformation of the central structure,^36^ for each pH value. The preparation of the pqr file was made using the in-house package meadTools (version 2.2),^33,35^ and the LPB and NLPB equations were solved with the APBS package.^40−42^ The atomic charges and radii were obtained from the GROMOS 54A7 force field as explained in ref (43). The molecular surface was defined with a solvent probe of radius 1.4 Å and a Stern layer of 2.0 Å, whereas the dielectric constant was set to 4.0 for the molecular interior and 80 for the solvent. The temperature was set to 300 K and the ionic strength to 0.1 M. For the monomer, a coarse grid of 192 Å with a 2 Å spacing and a fine grid of 96 Å with a 1 Å spacing were used. For the dimer, a coarse grid of 256 Å with a 2 Å spacing and a fine grid of 128 Å with a 1 Å were used. These settings were the same used in the CpHMD simulations, with exception of the protein dielectric constant and grid dimensions. For further details on PB calculations, see the Supporting Information.

### 2.3 Ion Excess

The excess of ions of each species *i* in a given region  is defined as the difference between the number of ions *N*_*i*_ found in that region and the number  that would be found in an equal-volume region in the bulk:^6^
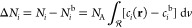
3Thus, the ion excess can be directly computed from the concentration grid maps obtained from either the MD simulations or the PB calculations (see Section 2.2). In particular, we will compute ion excess values for regions consisting of the set of grid bins that are within a given distance from the closest protein atom (see Section 2.4).

The ion excess for the whole MD simulation box follows directly from the ion-addition protocol used when building the system. As indicated in ref (23), the numbers of cations *N*_+_ and of anions *N*_–_ were chosen as the ones that result into charge neutrality and an ionic strength equal to the bulk value *I*, which for a 1:1 salt like NaCl corresponds to simultaneous satisfy the equations
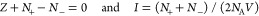
4where *Z* is the average protein charge (in protonic units) and *V* is the volume occupied by the solvent in the simulation box; the computed real values were then rounded to the nearest integers. The *N*_+_ and *N*_–_ values thus obtained satisfy the two conditions only approximately during the constant-pH MD simulations, because *Z* and *V* would fluctuate, but the conditions are expected to hold on average to a very good approximation; e.g., the average neutrality was kept within ±0.5 (except for the monomer at pH 5, with charge 1.2), as shown in Table S1 in the Supporting Information. Since the ionic strength for a region with volume *V* in the bulk is , with , we get from the conditions in eq 4 that the neutrality condition can be written as *Z* + Δ*N*_+_ – Δ*N*_–_ = 0, and that
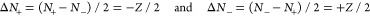
5Therefore, the ion excess values for the whole MD simulation box can be computed directly from the numbers of ions or from the protein charge, even though these relations should hold only approximately.

### 2.4 Ion–Protein Distances

All ion–protein distances used in the analyses were computed with respect to the closest protein atom. In the case of explicit MD ions, distances are computed between each ion and the closest protein atom in that simulation snapshot. In the case of ion concentration maps (Section 2.2), distances are computed between each bin of the map and the closest atom of the respective central structure.^36^

Radial distribution functions (RDFs) for the ions were also computed with respect to the closest protein atom. Since the protein irregular shape prevents a proper estimation of radial bin volumes, we used a nonstandard RDF defined in terms of molalities, instead of the densities traditionally used in the theory of liquids. The ensemble-averaged histogram of the number of particles of species *i* at a distance *r* from the closest protein atom is defined as
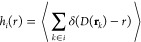
6where the angle brackets denote ensemble averaging, the sum extends over all particles *k* of species *i*, δ is the Dirac delta function, and *D*(**r**_*k*_) is the distance between a particle located at **r**_*k*_ and its closest protein atom. The molality of species *i* at a distance *r* from the closest protein atom is then *m*_*i*_(*r*) = *h*_*i*_(*r*)/(*h*_water_(*r*)*M*_water_), where *M*_water_ is the molar mass of water, and the molality-based RDF is defined as , with  being the bulk molality of species *i*. In practice, the discretized counterpart of *g*_*i*_(*r*) for a bin centered at *r* was computed using the discretized counterparts of the histograms, which were directly obtained with the GROMACS rdf tool with options -ref and -surf;^44^ since this use of the tool only works for atoms, the histogram for water was computed as the average of its three atom-based histograms, i.e., as *h*_water_(*r*) = (*h*_OW_(*r*) + *h*_HW1_(*r*) + *h*_HW2_(*r*))/3. Because it does not require any volume estimation, this molality-based RDF is unaffected by the protein irregular shape, being thus properly normalized.

## 3 Results and Discussion

### 3.1 Bound Ions

No site-bound ions were observed in the MD simulations, in agreement with the fact that no tightly associated ions are found in experimentally derived structures.^45−47^

**Figure 1:**
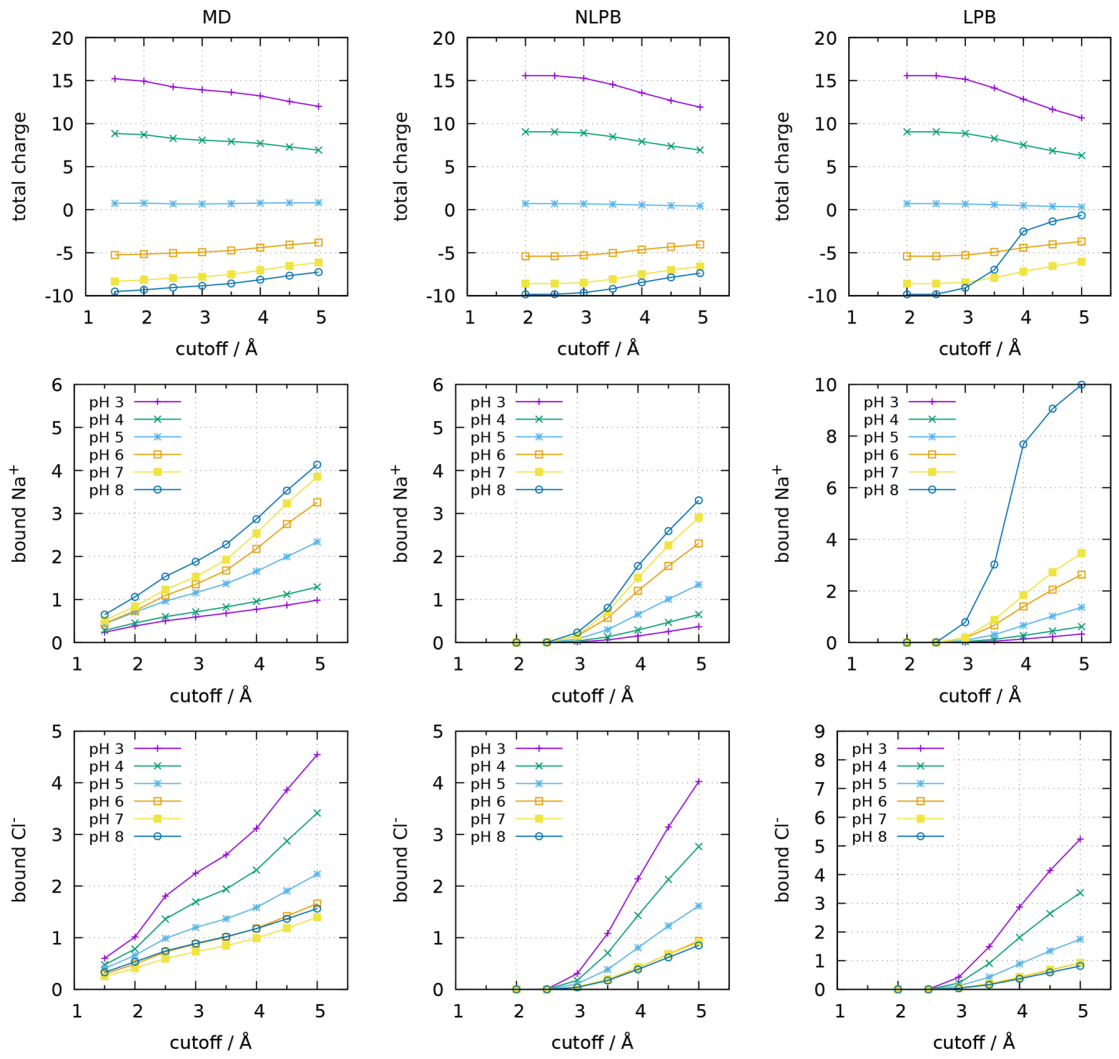
Protein total charge (top), number of bound Na^+^ (middle), and number of bound Cl^–^ (bottom) considering different cutoff distances from the closest protein atom, for the monomeric form of BLG at different pH values. Different methods are compared: on the left (MD), the number of bound ions is the average number of ions found within a cutoff distance from the protein during the CpHMD simulations; on the middle (NLPB), the number of bound ions of each ion type *i* is calculated as the integral of the NLPB-computed concentration map *c*_*i*_ (see Section 2.2) within a given cutoff distance from the protein; and on the right (LPB), the number of bound ions of each ion type *i* is calculated as the integral of the LPB-computed concentration map *c*_*i*_ within a given cutoff distance from the protein. The protein total charge is the net charge of the ionized residues plus the bound ions.

**Figure 2:**
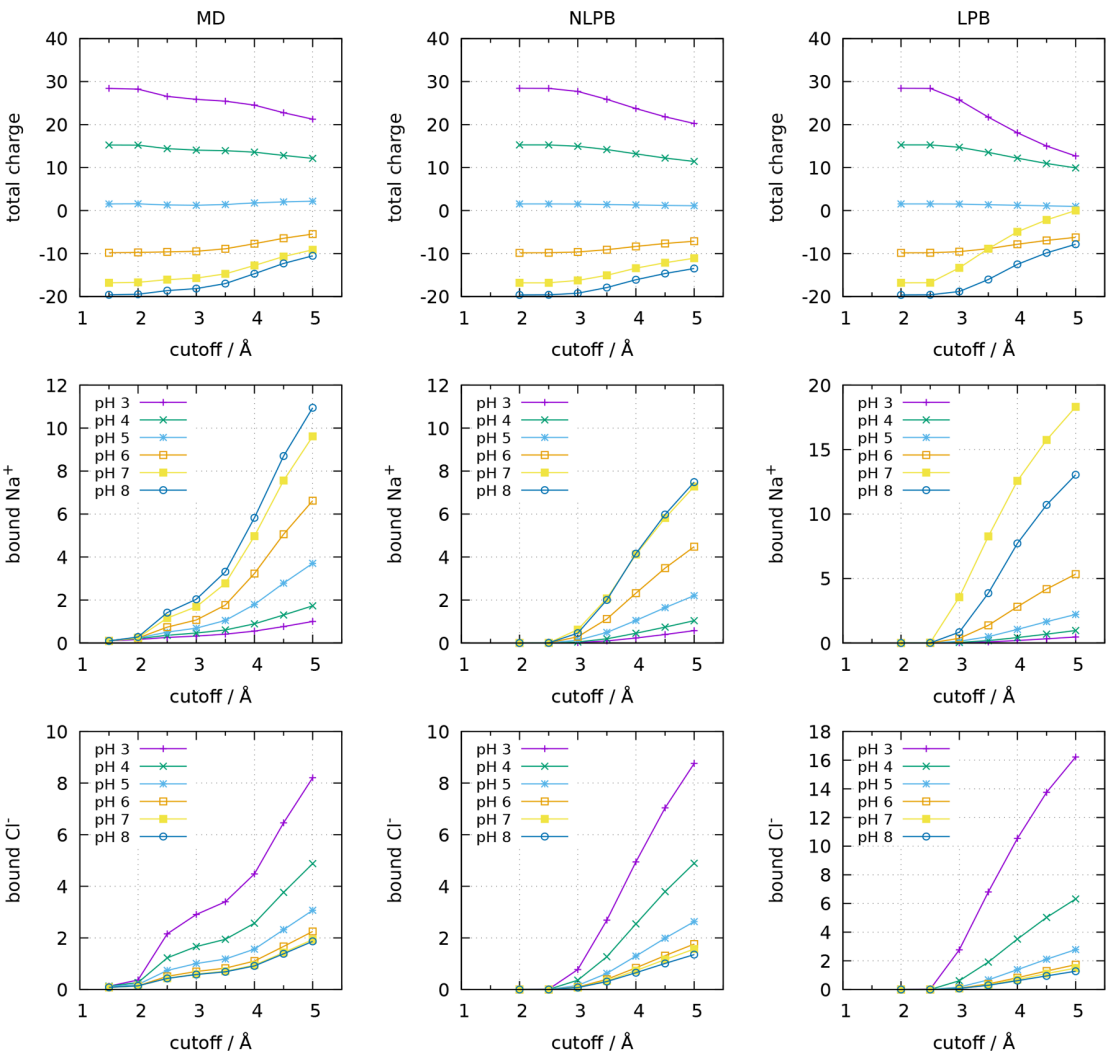
Protein total charge (top), number of bound Na^+^ (middle), and number of bound Cl^–^ (bottom) considering different cutoff distances from the closest protein atom, for the dimeric form of BLG at different pH values. Different methods are compared: MD, NLPB, and LPB. For details, see the caption of Figure 1.

We calculated the number of Na^+^ and Cl^–^ ions territorially bound to BLG and the resulting protein net charge (Figures 1 and 2). Since there is no obvious cutoff distance below which to consider an ion bound to the protein, we tested several distances up to 5 Å from the closest protein atom (see Section 2.4). In addition, the determination of the number of bound ions was done by three different alternative approaches: first, we used the CpHMD trajectories to count the number of ions below each cutoff distance; second, we used Na^+^ and Cl^–^ concentration maps calculated with the NLPB equation, as explained in Section 2.2; and, third, we did the same using the LPB equation. Whereas the first two approaches were in good agreement, the use of the LPB equation produced dissonant results at pH 3 and 8 for the monomer and at pH 3, 7, and 8 for the dimer (the reader may even notice the use of a different y scale for this method in Figures 1 and 2). The use of LPB electrostatic potentials seems to introduce some artifacts when calculating ionic concentrations with eq 2, which may be due to the fact that, since the LPB equation is obtained from discarding all terms beyond the second in the Taylor expansion of the exponential,^38^ going back to the full exponential function introduces an inconsistency.

On the other hand, the good agreement between the results obtained from the CpHMD trajectory and from the ion concentration maps calculated with NLPB suggests that these average quantities could be estimated even when no ions were included in the MD simulations. To check this, we calculated new ion concentration maps using the NLPB equation with a central structure and average charges from CpHMD simulations performed without explicit ions (the preliminary simulations in ref (23)). The results are, indeed, very similar, as shown in Figure S1, which is consistent with previous observations that the stochastic titration method is not significantly affected by the explicit inclusion of ions.^23,24^

**Figure 3:**
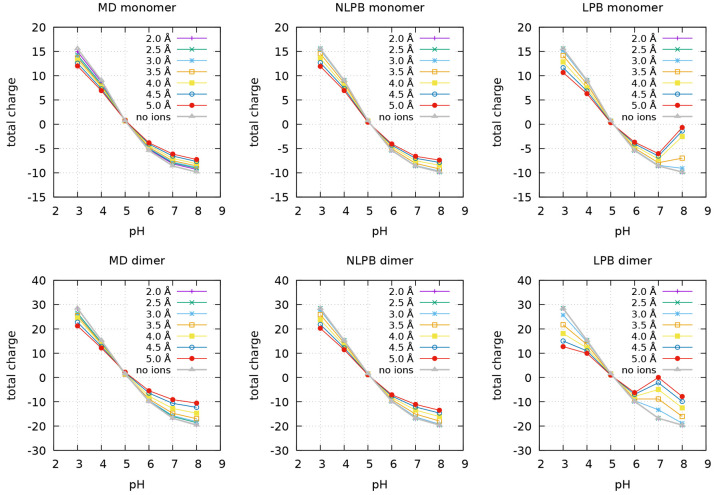
Protein total charge at different pH values, for the monomer (top) and dimer (bottom), using different cutoff distances from the closest protein atom. Different methods are compared: MD, NLPB, and LPB. This figure reproduces the results shown in the top rows of Figures 1 and 2 with an alternative representation. For details, see the caption of Figure 1.

**Figure 4:**
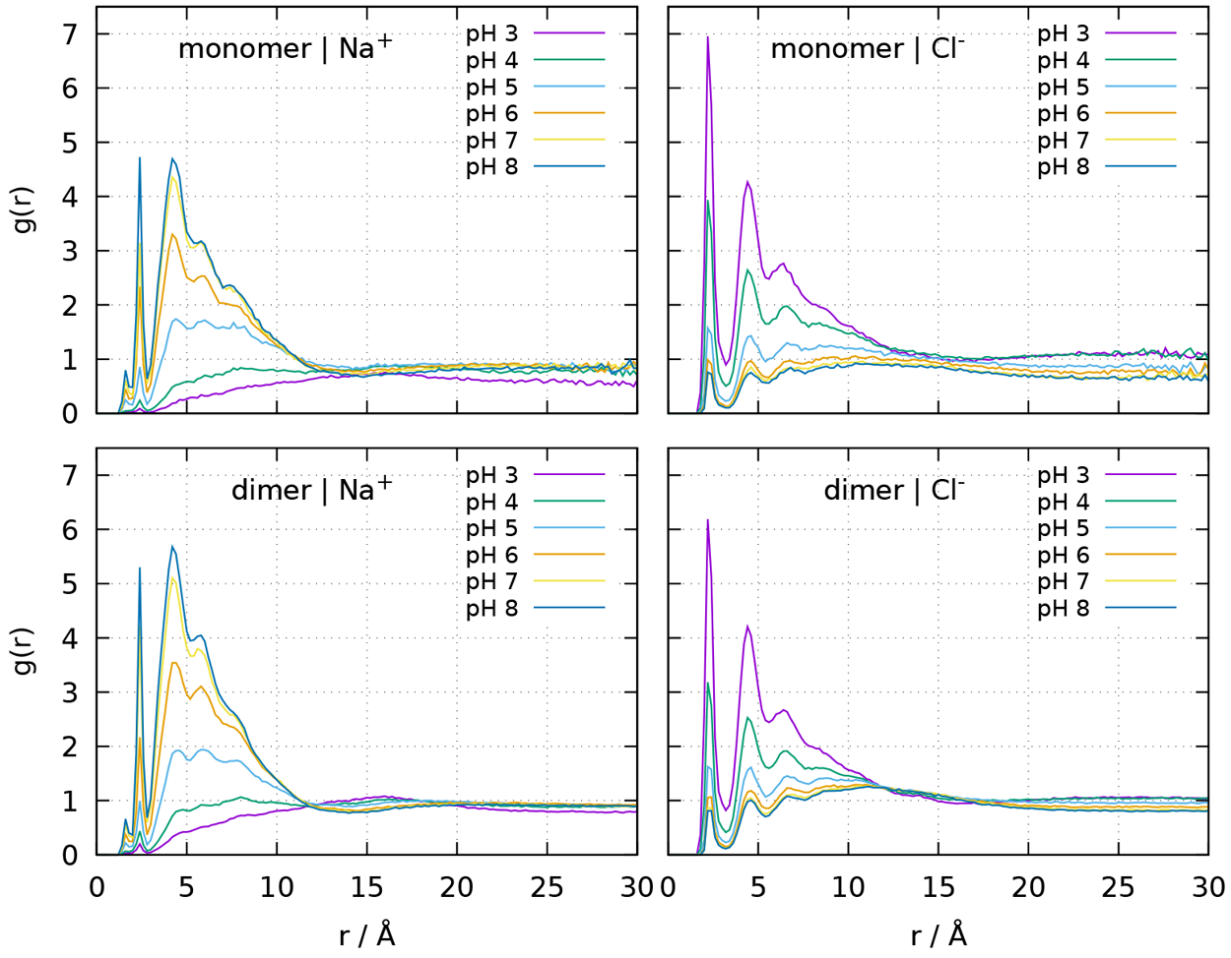
Radial distribution function of Na^+^ (left) and Cl^–^ (right) around the monomeric (top) and dimeric (bottom) forms of BLG at different pH values, computed in terms of molality and using ion distances to the closest protein atom (see Section 2.4).

Figure 3 shows how BLG total charge is varying with pH at the tested cutoff distances. The charge values obtained from the CpHMD trajectories and from the use of the NLPB equation are, in general, very similar, particularly at lower pH values. The results obtained with the LPB equation are also shown to reinforce their obvious discrepancy. There is no clear-cut criterion to decide whether a particular ion is territorially bound or not at a given instant, but it seems sensible to require that, if some ions are effectively bound, even if weakly, they should contribute to the first clearly discernible peaks in their radial distribution functions (RDFs), which are present up to a distance somewhere between 3.5 and 5.0 Å, depending on whether we consider the second large peak or not (Figure 4). The monomer total charge at pH 3, using the CpHMD trajectories and the 3.5 to 5.0 Å cutoffs, is 12.0–13.6, which is in excellent agreement with the value of 13.7 ± 1.5 measured in similar conditions (dilute solution, same ionic strength and pH) with membrane-confined electrophoresis using a Debye–Hückel–Henry correction for the charge^48,49^ by Thomas Laue and co-workers (personal communication). Therefore, this criterion based on the RDF peaks seems to capture the molecular entity probed by electrophoretic experiments. The ion–protein RDFs obtained here for Na^+^ and Cl^–^ are similar to others obtained at ionic strength 0.1 M using standard MD,^20−22^ even though ours include also the effect of the ionization equilibrium of the protonatable groups of the protein. Interestingly, a similar cutoff distance (5.0 Å) was found to yield good ion binding predictions in a PB-based method.^15^ The explicit accounting of pH-dependent ion binding is expected to improve the prediction of other measurable properties, as recently illustrated with PB predictions of measured zeta-potentials.^50^

### 3.2 Distribution of Ionic Concentrations

So far, the analyses of ions have shown a good agreement between the CpHMD trajectories and the ion concentration maps obtained with the NLPB equation. In this subsection, ion concentration maps obtained from the ionic densities in the CpHMD trajectories (Section 2.2) are used to further compare the MD and NLPB-based analyses. More specifically, the dispersion of ionic concentration values and the spacial distribution of ionic concentrations are examined.

**Figure 5:**
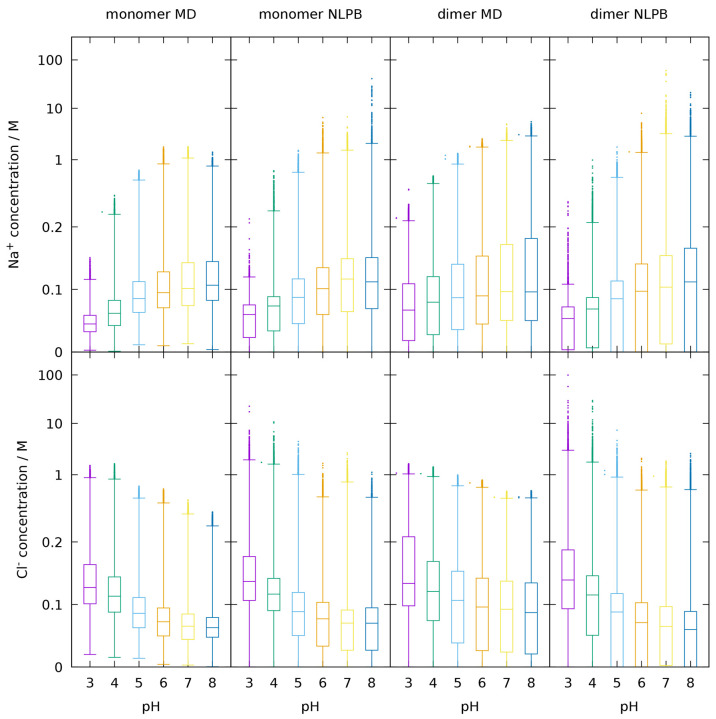
Boxplots of the Na^+^ (top) and Cl^–^ (bottom) concentrations found within a 15 Å distance from the closest protein atom, corresponding to the discrete concentration maps obtained from either the MD simulations or the NLPB calculations (see Section 2.2), for the monomer and dimer. The boxes delimit the interquartile range split in two by the median value, the whiskers enclose a 0.999 fraction of the concentration values, and outliers are depicted as points. The vertical scale is linear up to 0.2 M and logarithmic beyond that.

Figure 5 uses boxplots to represent the dispersion of Na^+^ and Cl^–^ concentrations at different pH values when using the maps obtained either with MD or NLPB. The analyses are performed within a cutoff of 15 Å from the protein due to the limited size of the monomer’s MD simulation box (see Section 2.2). Both sets of maps (MD and NLPB) present the expected trend of higher Na^+^ concentrations becoming more frequent as pH increases while the opposite is observed for Cl^–^. Although a large fraction of the data (0.999) is enclosed by the whiskers, outliers are also represented, and concentrations above 10 mM (note the nonuniform vertical scale) can be observed in the NLPB maps but not in the MD ones. In general, the NLPB concentration maps present higher dispersion and higher median values within the 15 Å radius. In addition, the dimer NLPB maps at some pH values present at least one-quarter of their space with concentration zero (since the boxes that enclose the interquartile region reach the zero lower limit), in contrast with the high concentration outliers also presented. A significant fraction of bins with 0 M concentration was expected due to the space occupied by the protein; whereas the PB model imposes a complete absence of ions below the Stern layer, the MM/MD model allows for ions to occasionally penetrate the interstices of the protein. In the monomer, at all pH values, the ions seem to sweep all space in the CpHMD trajectories, which, despite also being related to the flexibility of the protein, indicates occasional penetration of the protein interior, a fact confirmed by visual inspection.

**Figure 6:**
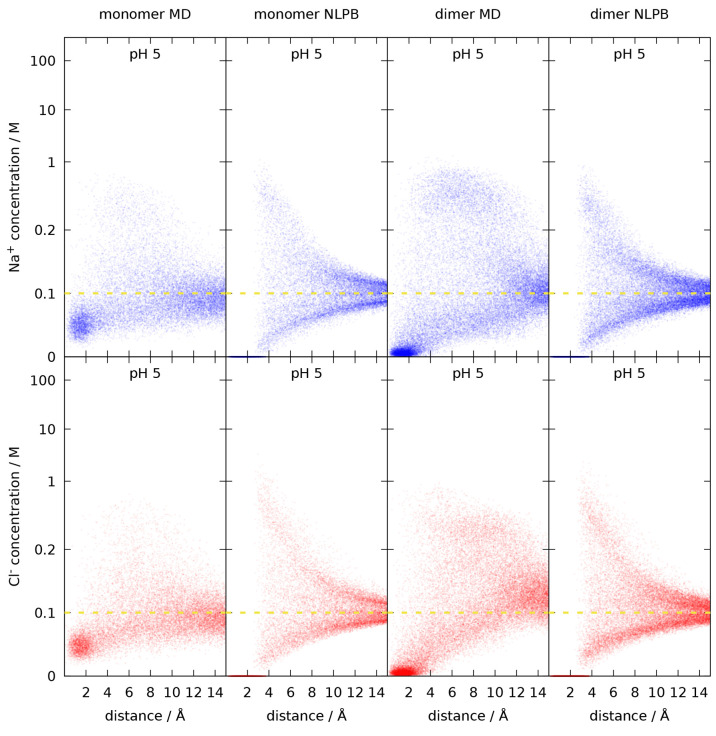
Scatter plots of Na^+^ and Cl^–^ concentrations versus the distance from the closest protein atom at pH 5, found within a 15 Å distance from the protein. The scatter plots obtained by using the ion concentration maps from the MD or from the NLPB calculations are shown, for the monomer and dimer. The yellow dashed line indicates the bulk ionic strength of 0.1 M. The vertical scale is linear up to 0.2 M and logarithmic beyond that.

This dispersion of concentrations can be further analyzed by depicting the map bins in a scatter plot of ion concentration versus distance to the protein, as shown in Figure 6 for pH 5 and in the Supporting Information for the other pH values (Figures S2–S7). In agreement with what was previously discussed, a cluster of low concentrations at very short distances is observed in the MD maps whereas at similar distances the concentrations are exactly zero in the NLPB maps. In most maps, a high dispersion is observed around a distance of 4 Å, sometimes even with some bimodal character, but the cloud of dots gradually funnels toward the value of the ionic strength as the distance increases. This effect is more marked in the NLPB maps, which display more symmetrical plots than the MD ones, as well as a higher dispersion at smaller distances that dissipates more abruptly with distance. The general effect of pH is the one that could be expected. At low pH, the scatter plots of the Na^+^ ion tend to occupy the region below the bulk ionic strength while, in the case of the Cl^–^ ion, the region above is occupied. Conversely, at higher pH, the scatter plots of the Na^+^ and the Cl^–^ ions tend to occupy, respectively, the regions above and below the bulk ionic strength, but this effect is less marked than at low pH. Around the isoionic point (pH 5), a roughly symmetrical shape in relation to the ionic strength axis is observed for both anions and cations. In the case of the NLPB plots there is also an inevitable relation between the Na^+^ and Cl^–^ maps, because eq 2 implies that, for the 1:1 salt being considered, *c*_+_/*I* = *I*/*c*_–_ (e.g., for *I* = 100 mM, a bin with *c*_–_ = 200 mM will have *c*_+_ = 50 mM); thus, the NLPB plots for Na^+^ and Cl^–^ show also a necessary “reciprocal symmetry” between themselves, which is not present in the MD case. Overall, these scatter plots indicate that the high dispersion of concentration values relates to the heterogeneity of ion occupation close to the protein surface, which is directly related to the local protein charge and highly dependent on pH. Thus, for example, even though Na^+^ plays the role of co-ion at pH 4, a population of bins with more than twice the bulk ionic strength can be observed between 4 and 10 Å for the Na^+^ map computed from the MD simulations of the dimer (Figure S3). This charge heterogeneity is most marked at pH 5, when the overall neutral protein consists of multiple positive and negative patches, which gives rise to the high dispersion already noted.

**Figure 7:**
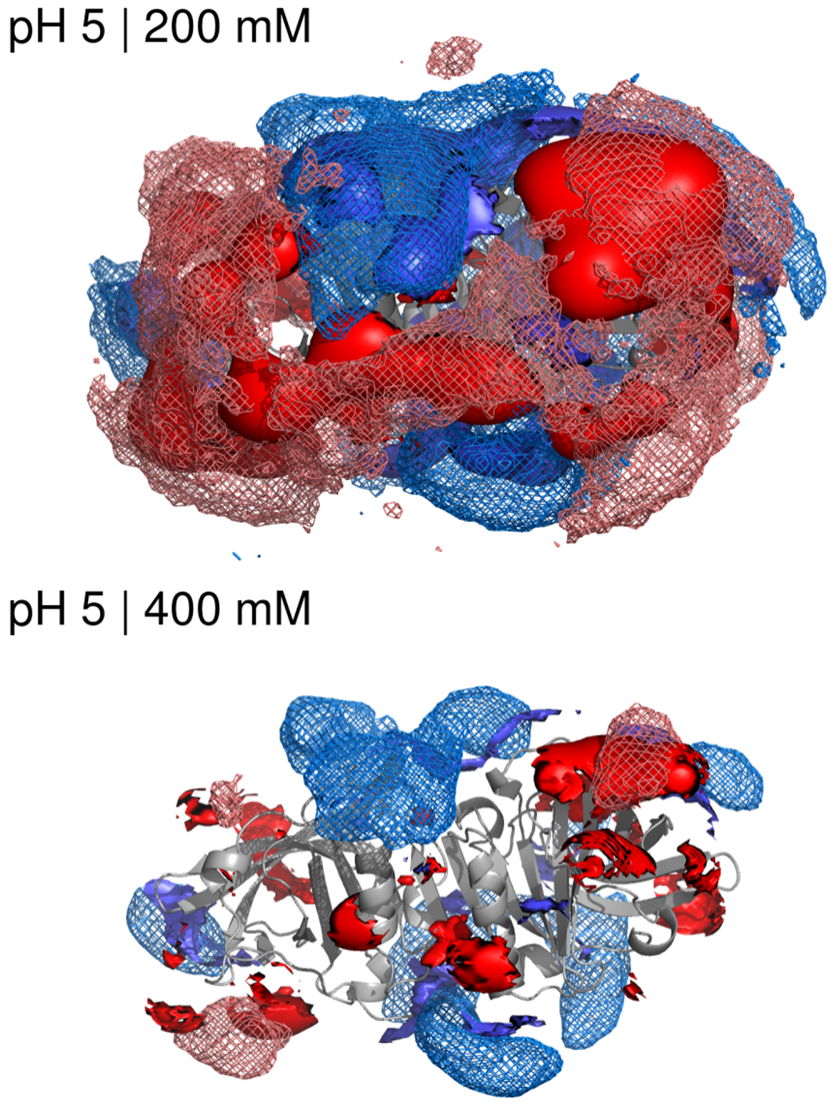
Ion iso-concentration contours of 200 mM (top) and of 400 mM (bottom) for Na^+^ (blue) and Cl^–^ (red) in the dimer at pH 5, obtained from either the MD (mesh contours) or the NLPB (solid contours) concentration maps.

The charge heterogeneity of the protein surface can be seen in detail using concentration iso-contours, as shown in Figure 7 for the dimer at pH 5 and in Supporting Information for the other pH values (Figure S8). The concentration contours obtained with PB and MD maps present a reasonable overall agreement for a contour level of 200 mM, as previously observed,^23^ but differences become evident with contours of 400 mM, with the PB contours more scattered and ragged than the MD ones. The PB concentration maps must follow the shape and charge details of the surface of the single protein structure used in the PB calculation, while the MD maps experience a “leveling” effect due to the structural and ionization fluctuations at the protein surface, with ions ending up being less localized/concentrated; this is probably one of the reasons why the differences between PB and MD become more evident with higher-concentration contours, which are closer to the protein surface. Moreover, although the ions in the MD simulations can approach and even occasionally penetrate the protein (see above), the protein structural fluctuations have also the average effect of “wiping off” the ions closest to the surface, which explains why the MD contours (especially the high-concentration ones) are generally farther from the protein than the PB ones. In addition, the MD-derived contours obviously reflect the presence of ion–ion correlations, which are necessarily absent from the PB mean field treatment. This shows that the common procedure of doing a PB calculation using a representative protein structure (e.g., the crystallographic one) can fail to properly capture the average ion distribution near the protein surface.

### 3.3 Total Charge and Ion Excess

The ion densities in the MD and PB analyses can also be compared in terms of the ion excess and the progressive neutralization of the system at increasingly larger regions surrounding the protein. In any case, we should note that the moderate MD box sizes used here, typical of most simulations, are known to lead to ion accumulation and depletion at large distances in the case of nucleic acid simulations.^13,26^ The fact that the tails of the RDFs in Figure 4 show some deviation from the exact value of 1 indicate a similar limitation in our simulations, which would necessarily affect the convergence of the ion excess. This can be circumvented by using very large boxes (e.g., big enough to enclose up to around 800 ions in the case of an RNA molecule with charge −30^26^), which is usually prohibitive. Therefore, one should keep in mind that this limitation is always present in typical simulations.

**Figure 8:**
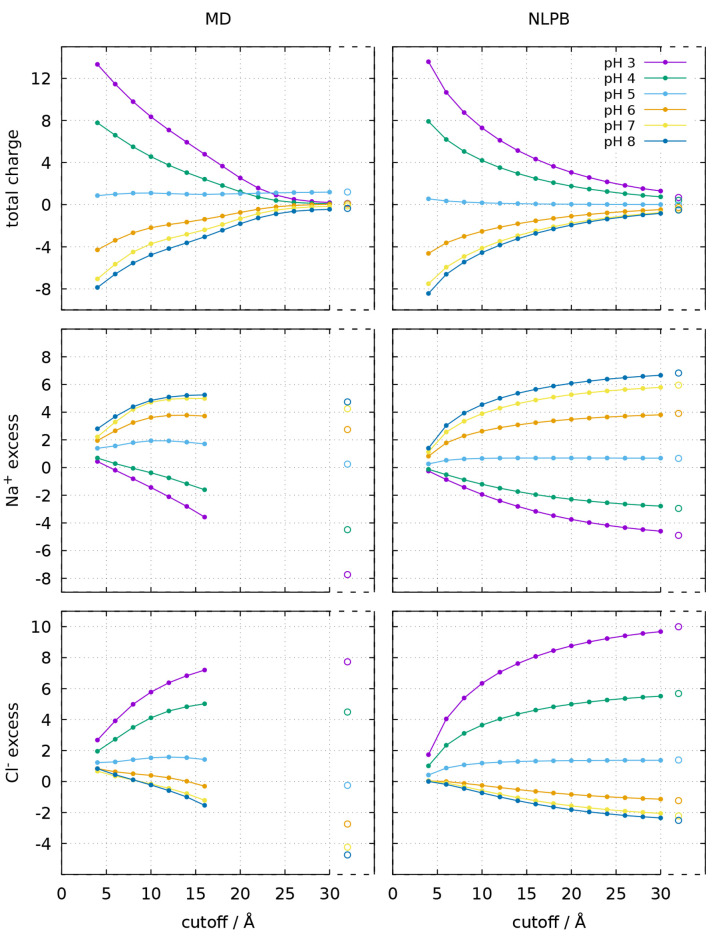
Total charge (top), Na^+^ excess (middle), and Cl^–^ excess (bottom) at different cutoff distances from the closest protein atom, calculated using the ion concentration maps obtained from the CpHMD trajectories (left) or calculated with the NLPB equation (right), for the monomer. The total charge is the net charge of the protein ionized residues plus the ions within the cutoff. The large empty circles indicate the values found, respectively, in the whole MD box or PB grid; in the MD case, these values are the average of the fluctuating charge of the whole box, which ensures only approximate neutralization (see Section 2.3 and Table S1). The ion excesses in the whole MD box were calculated as Δ*N*_+_ = (*N*_+_ – *N*_–_)/2 and Δ*N*_–_ = −Δ*N*_+_ (see Section 2.3). Due to the mismatch between the periodic nonrectangular box of the MD simulations and the nonperiodic rectangular grid of the concentration maps (see Section 2.2), distances above 16 Å were not analyzed for the MD ion excesses; but the MD total charge is still shown beyond that distance, as the whole box becomes enclosed within the cutoff.

**Figure 9:**
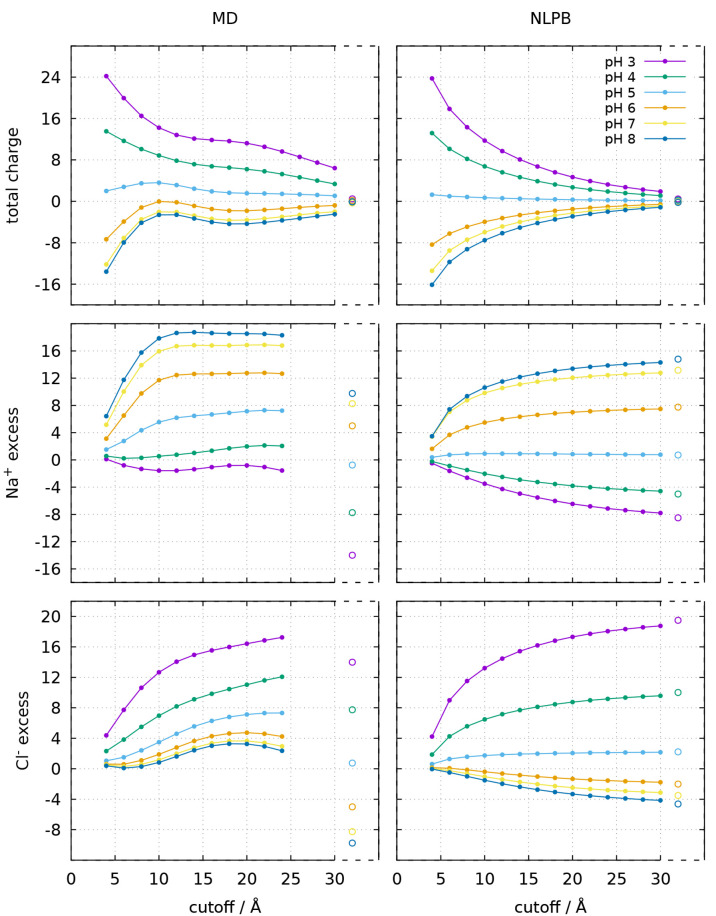
Total charge (top), Na^+^ excess (middle), and Cl^–^ excess (bottom) at different cutoff distances from the closest protein atom, calculated using the ion concentration maps obtained from the CpHMD trajectories (left) or calculated with the NLPB equation (right), for the dimer. See caption of Figure 8 for further details. A maximum bin–protein distance of 25 Å was used when computing the MD ion excesses for the dimer.

**Figure 10:**
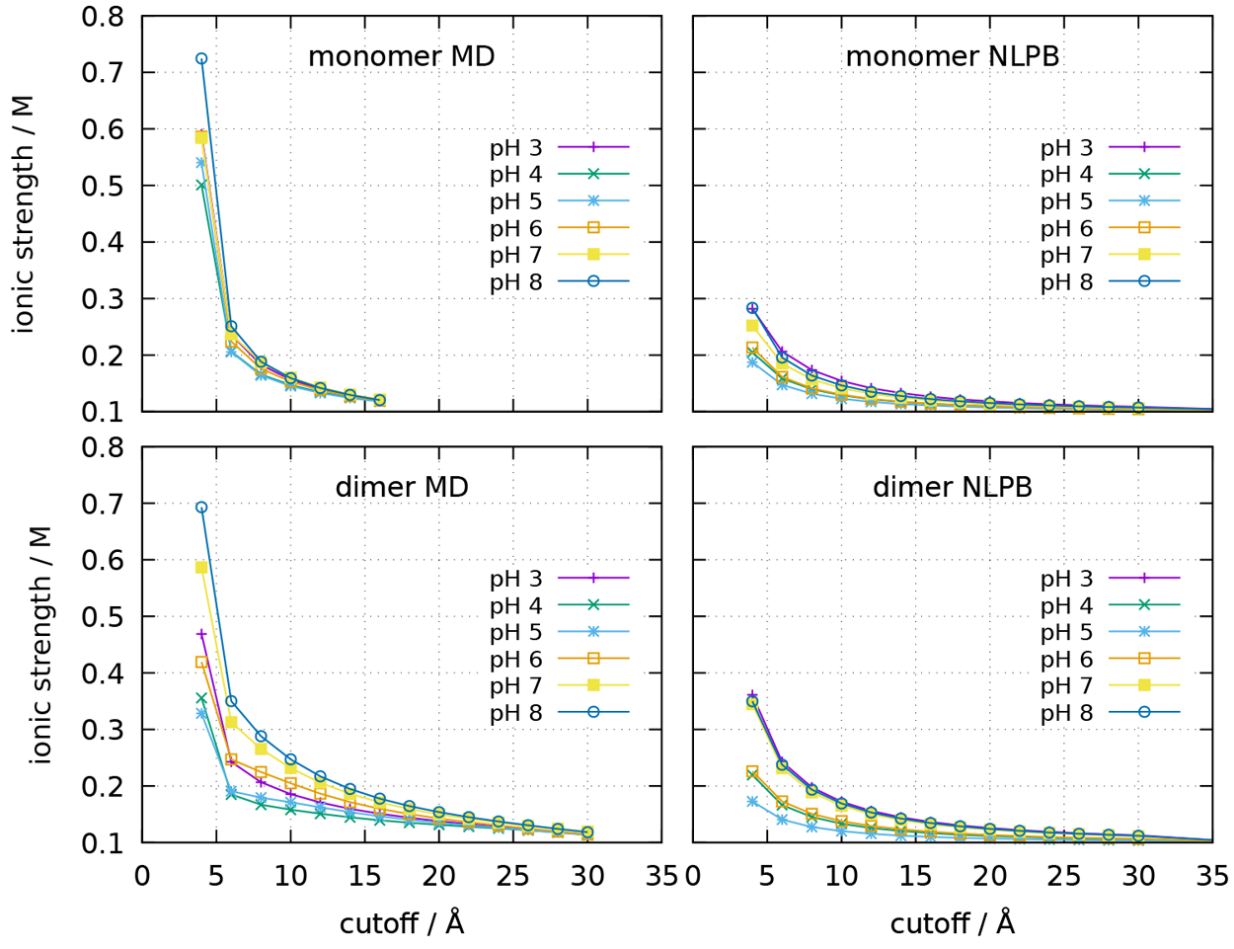
Ionic strength calculated at different cutoff distances from the closest protein atom, using the ionic concentration maps obtained from the MD simulations (left) or from the NLPB calculations (right), for the monomer (up) and dimer (bottom).

In Figures 8 and 9, the total charge, Na^+^ excess, and Cl^–^ excess within a cutoff distance from the protein are shown for the ion concentration maps obtained from MD and the ones obtained from NLPB (see Section 2.3). The total charge is properly converging to values close to zero in both sets of maps. In the MD case, box neutralization depends on the a priori estimation of the average protein charge and the number of ions accordingly added to the box (Section 2.3); though this estimation tends to be good, it may result in some small deviations. In the NLPB case, complete neutralization may not be observed in smaller grids like the one used for the monomer (96 Å), but in the larger dimer grid (128 Å) the total charge is very close to zero even at pH 3 and 8, despite the protein having twice the charge. In general, for both the total charge and the ion excess, there is a good agreement between the pH profiles of the MD and NLPB analyses, but the NLPB curves are more regular and smoother. Another noteworthy difference is that the curves of the ion excess are converging to different values in the two methods. As pointed out in Section 2.3, the conditions imposed in the MD simulations imply that the Na^+^ and Cl^–^ excesses in the whole box are symmetrical and have an absolute value equal to the protein half-charge *Z*/2 (Δ*N*_±_ = ∓*Z*/2), but this same condition is not verified in the NLPB grids, which converge to higher values (i.e., counterion excesses become more positive and co-ion excesses become less negative). However, the fact that the sum of Na^+^ and Cl^–^ excesses is not converging to zero does not affect the convergence of the ionic strength to the bulk value, as can be verified in Figure 10. This may signify that although the ionic strength rapidly converges to the bulk value in the NLPB model, it only reaches its exact value at infinity. In contrast, the ionic strength in the MD box presents much higher values near the protein and seems to converge more slowly, even though it necessarily yields the exact value when the whole box is considered (see section 2.3).

Although no experimental determinations of ion excess have been done for BLG, it is instructive to compare our results with those recently obtained for other proteins by Iwahara and co-workers using NMR-based quantification.^8^ This study determined the anion (acetate) excess at pH 7.5 for three proteins: 6.7 for Antp homeodomain (Antp HD), 4.1 for bovine pancreatic trypsin inhibitor (BPTI), and 0.1 for ubiquitin (±0.1 in all cases). The total charge *Z* of each of these proteins at pH 7.5, estimated from the typical p*K*_a_ values of the protein ionizable groups,^8^ should be respectively around 12, 6 and 0, meaning that, as noted by the authors, the measured anion excess follows closely the half-charge relation Δ*N*_–_ = +*Z*/2 indicated above. The authors interpreted their experimental ion excess values as corresponding to the whole ion atmosphere, understood in this context as the region around the protein which is large enough so that its ions cancel the protein charge,^1,8^ which, interestingly, is one of the conditions imposed on our MD simulations (eq 4); so, our MD simulation box is in a sense trying to play the role of the whole ion atmosphere, despite its perhaps limited size. The authors of this study also used an NLPB model to compute the ion excesses within the whole grid, for a 1:1 salt, obtaining^8^ Δ*N*_–_ = 8.2 and Δ*N*_+_ = −3.8 for Antp HD, Δ*N*_–_ = 3.5 and Δ*N*_+_ = −2.5 for BPTI, and Δ*N*_–_ ≈ Δ*N*_+_ ≈ 0 for ubiquitin. Thus, as *Z* increases, their NLPB-computed ion excesses also increase not only relative to the half-charge ±*Z*/2, like we observe, but also relative to the experimental values (see Figure S9). In addition, their NLPB-computed excesses, obtained with very large grids, satisfy the neutrality relation *Z* + Δ*N*_+_ – Δ*N*_–_ = 0, like we observe more approximately with our smaller grids. Overall, these considerations indicate that our protocol for ion addition to the MD simulation box is in line with the currently available experimental results and is perhaps more sound than the NLPB approach, which may overestimate ion excess values.

## 4 Conclusions

In this work, we have investigated the binding and distribution of ions around BLG using CpHMD simulations of the monomeric and dimeric forms, from pH 3 to 8.^23^ The protocol of adding ions to the MD box, namely the requirements of approximate charge neutrality and ionic strength equal to that in the bulk, imposes a few conditions to be observed in the whole box, including the condition that the absolute value of the ion excess should be half the protein charge. This half-charge relation was also observed for three other proteins in a recent experimental study,^8^ which suggests that it may be a general feature of the ion atmosphere around proteins, thus supporting our adopted MD protocol.

The MD results were compared with a PB model, and both the LPB and NLPB equations were tested. The three approaches were used to estimate territorially bound ions and, while MD and NLPB were in general good agreement, LPB produced dissonant results, likely due to the theoretical inconsistency that it introduces in the calculation of the concentration maps; thus, the remaining analyses using the PB model were done with the nonlinear form of the equation. The protein total charge (including territorially bound ions) estimated using the CpHMD simulations was in excellent agreement with the value obtained from membrane-confined electrophoresis (personal communication by Thomas Laue).

Several analyses of the ion concentration maps obtained with MD and NLPB at the different pH values were performed, which included boxplots representing the dispersion of concentration values, scatter plots of concentration versus distance, inspection of iso-concentration contours around the protein, convergence of ion excess, and convergence of ionic strength. The two approaches presented coherent results regarding the expected trends for Na^+^ and Cl^–^ at the different pH values, besides correctly converging to the bulk ionic strength. Despite an overall good agreement, MD and NLPB differed in several details. For instance, the spatial localization of high iso-concentration contours did not coincide in several examples, bins of very high concentration at short distances from the protein were more common in NLPB, and, overall, NLPB analyses produced more regular and symmetrical patterns. A most striking difference was that the NLPB-computed ion excesses converged to values higher than the MD-computed ones, with the difference increasing with protein charge; a similar trend was observed in relation to experimental values,^8^ suggesting that the NLPB model may induce an overestimation of counterion excess and an underestimation of co-ion exclusion. Nonetheless, the overall good agreement between MD and NLPB indicates that, unless detailed aspects are of concern, the NLPB model probably provides a reasonable and computationally inexpensive route to study ion behavior around proteins, for which it may fare better than for the high charge density of nucleic acids.

Overall, this study shows that CpHMD simulations can provide a detailed characterization of how ions bind to and distribute around proteins as a function of pH. In addition to reflecting the protein flexibility and ion–ion correlations absent from PB models, these simulations also capture the effect of the acid–base equilibrium of the protein ionizable groups, not present in standard MD. Moreover, given the apparently good performance of our ion-addition protocol, it may be helpful in the context of other CpHMD methods in which the treatment of ions plays a major role.^25,26^

## Supporting Information Available

Details of PB calculations and CpHMD simulations; scatter plots of Na^+^ and Cl^–^ concentrations versus the distance from the protein; ion iso-concentration contours for Na^+^ and Cl^–^ around BLG dimer; and published experimental and NLPB-computed ion excess data versus protein charge, for Antp homeodomain, bovine pancreatic trypsin inhibitor, and ubiquitin (PDF)
