# Supplementary material for: Computational Study of the pH-Dependent Ionic Environment around β-Lactoglobulin
Source: J Phys Chem B. 2022 Nov 2;126(45):9123–36. doi: 10.1021/acs.jpcb.2c03797 (PMC9776516; doi:10.1021/acs.jpcb.2c03797)
Supplement: Supplementary file 1 — jp2c03797_si_001.pdf [file jp2c03797_si_001.pdf]

Supporting Information: Computational Study of the pH-Dependent Ionic Environment Around β-Lactoglobulin

Lucie da Rocha, António M. Baptista,* and Sara R. R. Campos*

Instituto de Tecnologia Química e Biológica António Xavier, Universidade Nova de Lisboa, Av. da República, 2780-157 Oeiras, Portugal

E-mail: baptista@itqb.unl.pt; scampos@itqb.unl.pt

Phone: +351-21-4469619; +351-21-4469619

List of Tables

S1 CpHMD conditions. S-4

List of Figures

S1 Protein total charge, number of bound Na^+, and number of bound Cl^- at different cutoff distances, estimated from ion concentration maps obtained with the NLPB equation using average charges and central structure from CpHMD simulations performed in the absence of ions. S-5

S2 Scatter plots of Na^+ and Cl^- concentrations vs the distance from the protein at pH 3. . . . . S-6

S3 Scatter plots of Na^+ and Cl^- concentrations vs the distance from the protein at pH 4. . . . . S-7

S4 Scatter plots of Na^+ and Cl^- concentrations vs the distance from the protein at pH 5. . . . . S-8

S5 Scatter plots of Na^+ and Cl^- concentrations vs the distance from the protein at pH 6. . . . . S-9

S6 Scatter plots of Na^+ and Cl^- concentrations vs the distance from the protein at pH 7. . . . . S-10

S7 Scatter plots of Na^+ and Cl^- concentrations vs the distance from the protein at pH 8. . . . . S-11

S8 Ion iso-concentration contours of 200 mM and of 400 mM for Na^+ and Cl^- in the dimer at different pH values, obtained from either the MD maps or the NLPB maps. S-12

S9 Experimental and NLPB-computed ion excess data versus protein charge, for Antp homeodomain, bovine pancreatic trypsin inhibitor and ubiquitin S-13

Poisson–Boltzmann conditions:

- Temperature: 300 K
- Ionic strength: 0.1 M
- Dielectric constant: 4 for protein; 80 for solvent
- Coarse grid:
  - Monomer: 192^3 Å^3 (spacing 2 Å)
  - Dimer: 256^3 Å^3 (spacing 2 Å)
- Fine grid:
  - Monomer: 96^3 Å^3 (spacing 1 Å)
  - Dimer: 128^3 Å^3 (spacing 1 Å)
- Stern layer (ionic radii): 2 Å

Table S1: CpHMD conditions.

	Monomer						
		Production	Image		Ions		Average
pH	Replicates	length (ns)	distance (nm)*	Waters	Na^+	Cl^-	net charge
3	4	280	3.12	9410–9724	10	25–26	0.07
4	4	280	3.16	9410–9723	13–14	22–23	0.04
5	4	280	3.21	9410–9724	18	17–18	1.20
6	4	280	3.24	9410–9724	21	15	0.09
7	4	280	3.23	9410–9724	22–23	13–14	−0.10
8	4	280	3.17	9410–9724	23	13	−0.35

	Dimer						
		Production	Image		Ions		Average
pH	Replicates	length (ns)	distance (nm)*	Waters	Na^+	Cl^-	net charge
3	8	515.20	4.69	36172–37590	54–56	82–85	0.46
4	8	560.00	4.72	36171–37590	60–63	76–78	−0.21
5	8	559.90	4.60	36171–37590	67–70	68–71	0.04
6	8	559.93	4.51	36172–37590	73–75	63–65	0.14
7	8	542.27	4.50	36171–37590	76–79	60–62	−0.30
8	8	544.83	4.59	36171–37590	77–80	58–61	−0.12

*Average value of the minimum distance between protein periodic images.

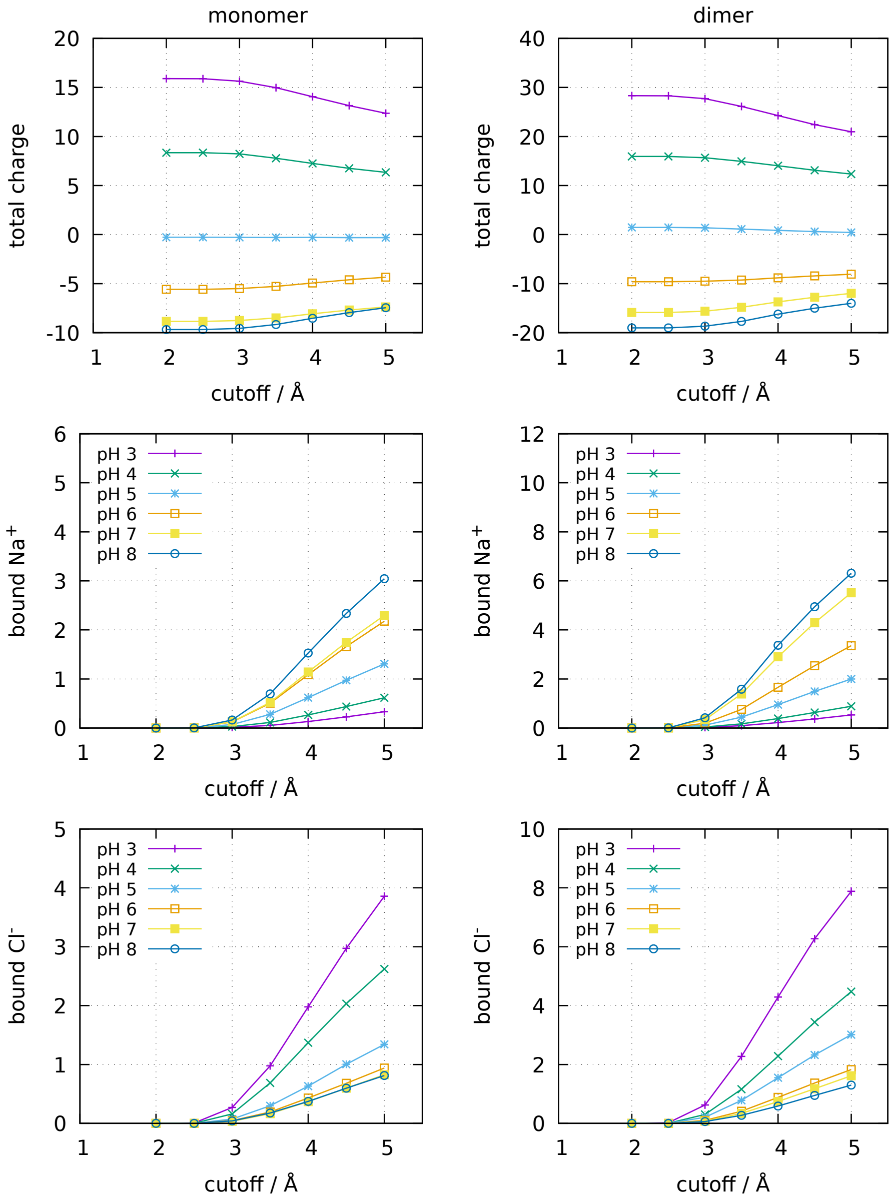

Figure S1: Protein total charge (top), number of bound Na^+ (middle), and number of bound Cl^- (bottom) at different cutoff distances, estimated from ion concentration maps obtained with the NLPB equation using average charges and central structure from CpHMD simulations performed in the absence of ions. The number of bound ions of each ion type i is calculated as the integral of the NLPB-computed concentration map c_i within a given cutoff distance from the protein. The protein total charge is the net charge of the ionized residues plus the bound ions.

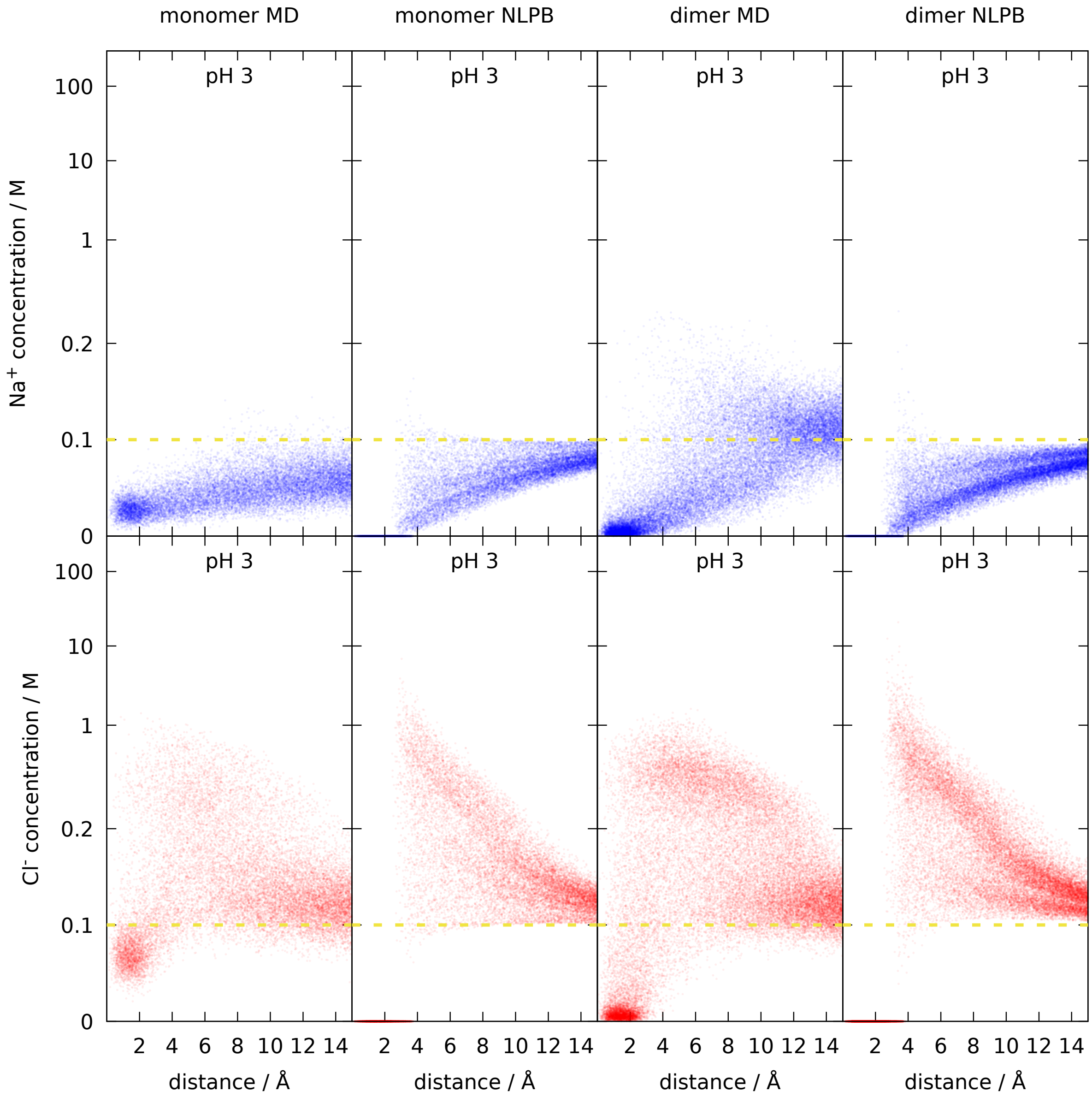

Figure S2: Scatter plots of Na^+ and Cl^- concentrations versus the distance from the closest protein atom at pH 3, found within a 15 Å distance from the protein. The scatter plots obtained by using the ion concentration maps from the MD or from the NLPB calculations are shown, for the monomer and dimer. The yellow dashed line indicates the bulk ionic strength of 0.1 M. The vertical scale is linear up to 0.2 M and logarithmic beyond that.

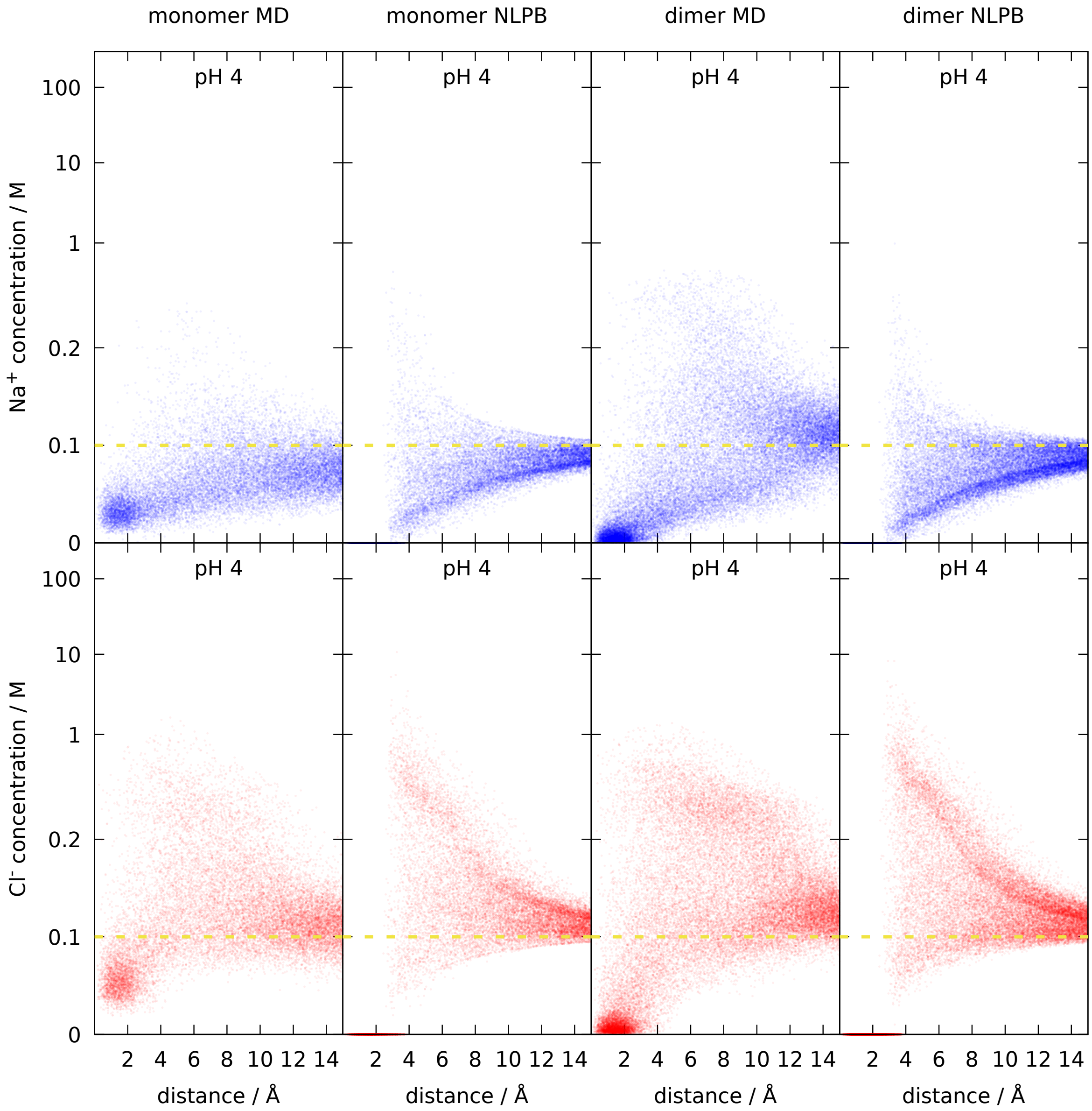

Figure S3: Scatter plots of Na^+ and Cl^- concentrations versus the distance from the closest protein atom at pH 4, found within a 15 Å distance from the protein. The scatter plots obtained by using the ion concentration maps from the MD or from the NLPB calculations are shown, for the monomer and dimer. The yellow dashed line indicates the bulk ionic strength of 0.1 M. The vertical scale is linear up to 0.2 M and logarithmic beyond that.

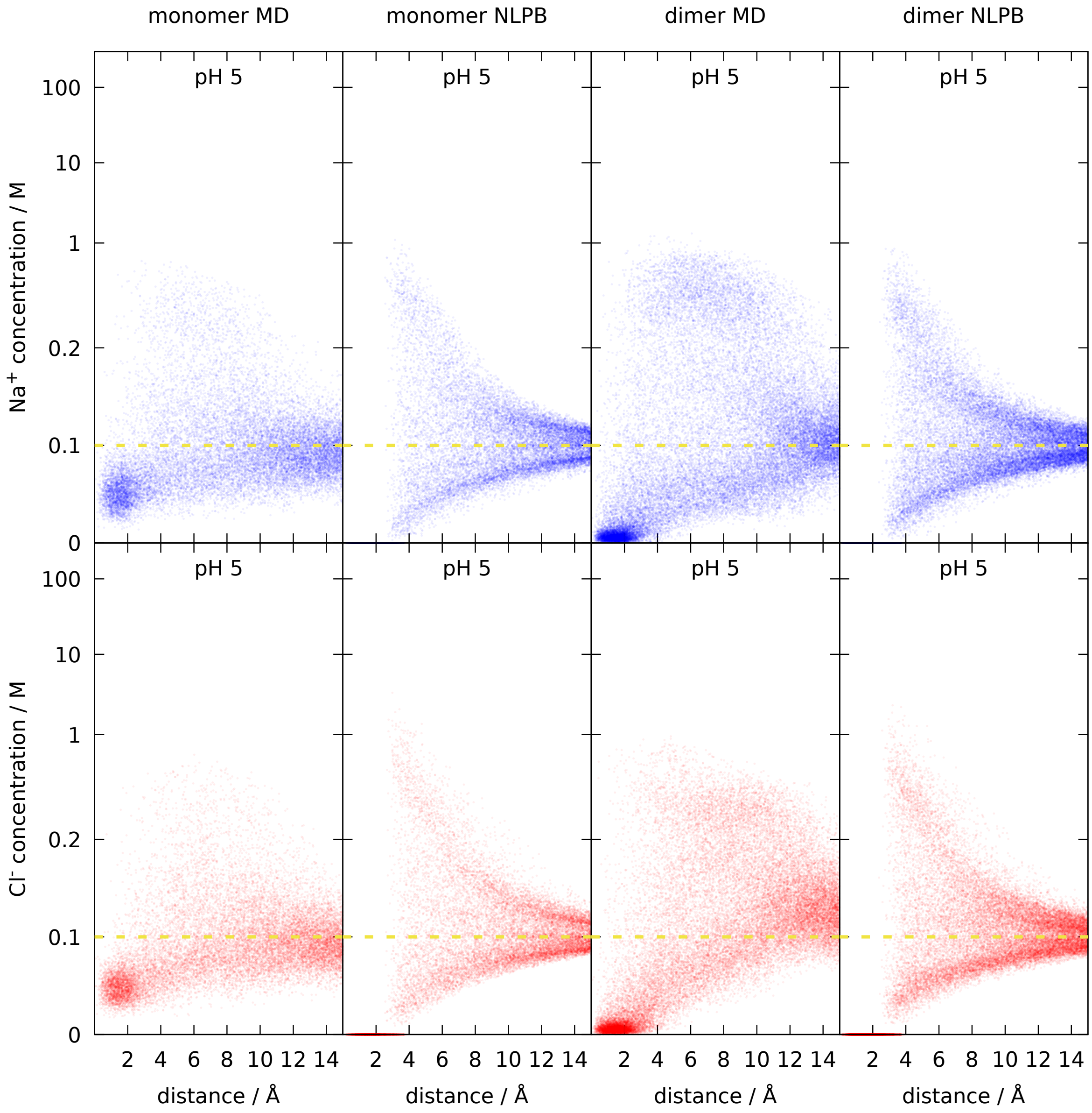

Figure S4: Scatter plots of Na^+ and Cl^- concentrations versus the distance from the closest protein atom at pH 5, found within a 15 Å distance from the protein. The scatter plots obtained by using the ion concentration maps from the MD or from the NLPB calculations are shown, for the monomer and dimer. The yellow dashed line indicates the bulk ionic strength of 0.1 M. The vertical scale is linear up to 0.2 M and logarithmic beyond that.

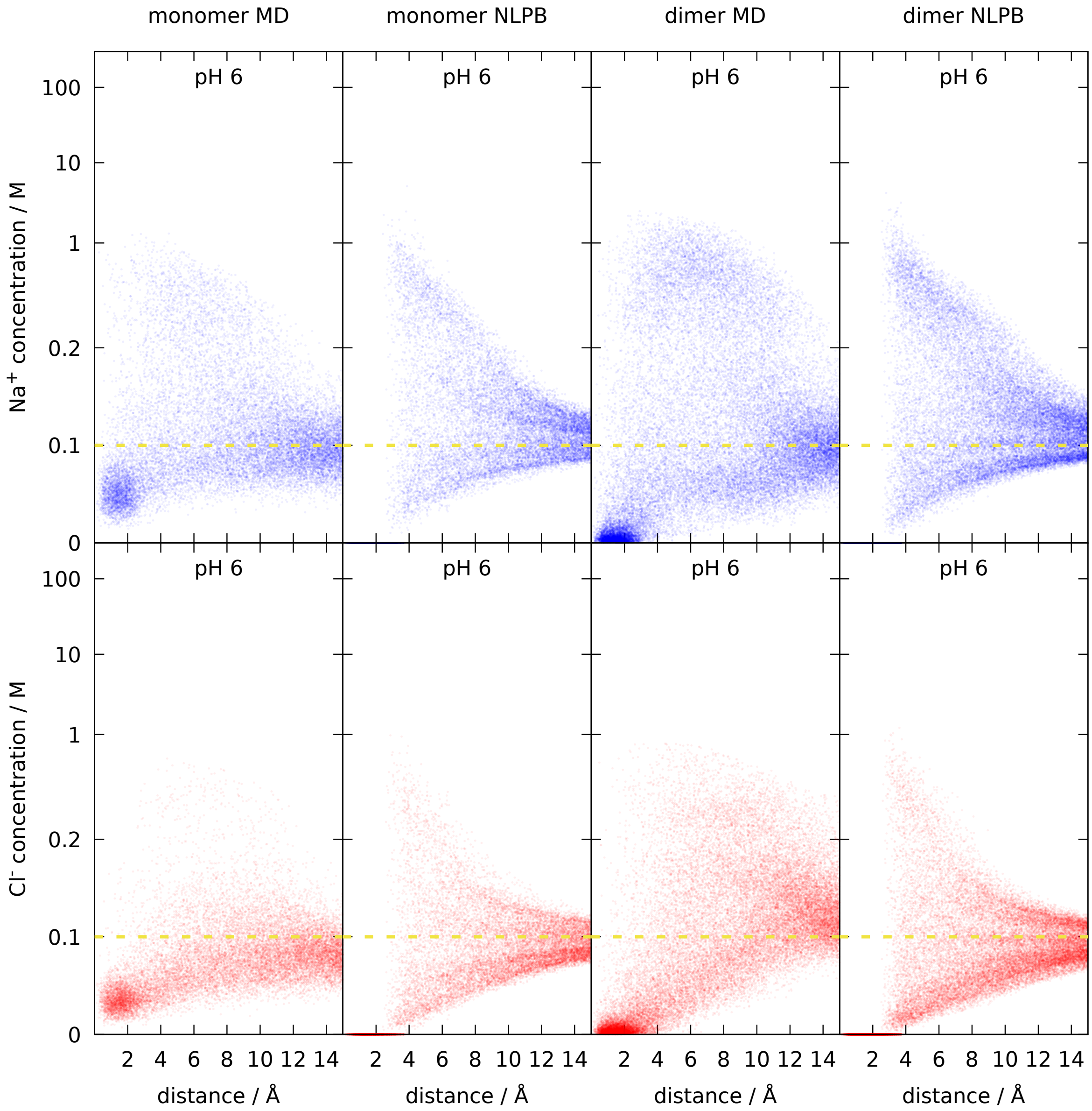

Figure S5: Scatter plots of Na^+ and Cl^- concentrations versus the distance from the closest protein atom at pH 6, found within a 15 Å distance from the protein. The scatter plots obtained by using the ion concentration maps from the MD or from the NLPB calculations are shown, for the monomer and dimer. The yellow dashed line indicates the bulk ionic strength of 0.1 M. The vertical scale is linear up to 0.2 M and logarithmic beyond that.

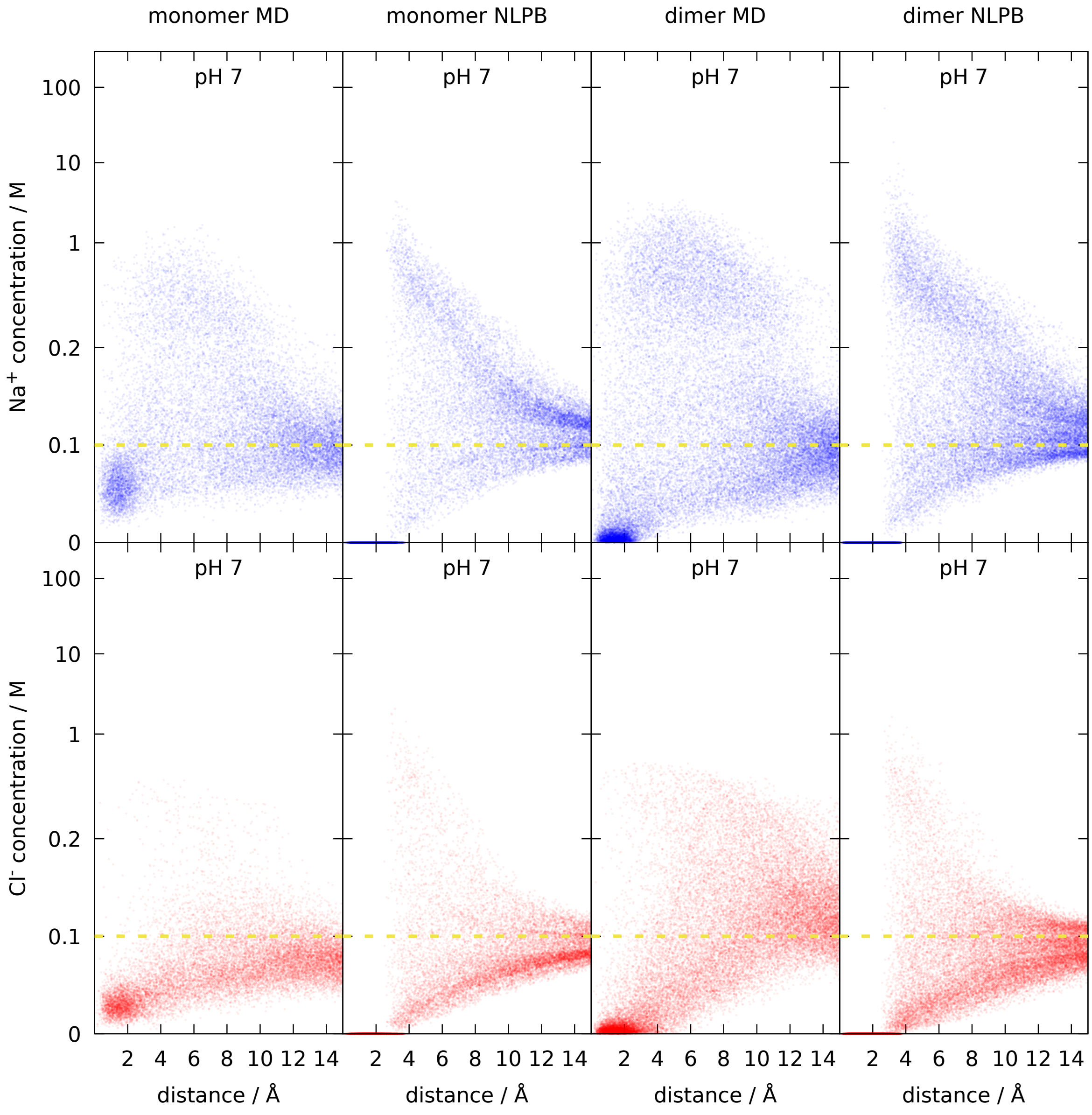

Figure S6: Scatter plots of Na^+ and Cl^- concentrations versus the distance from the closest protein atom at pH 7, found within a 15 Å distance from the protein. The scatter plots obtained by using the ion concentration maps from the MD or from the NLPB calculations are shown, for the monomer and dimer. The yellow dashed line indicates the bulk ionic strength of 0.1 M. The vertical scale is linear up to 0.2 M and logarithmic beyond that.

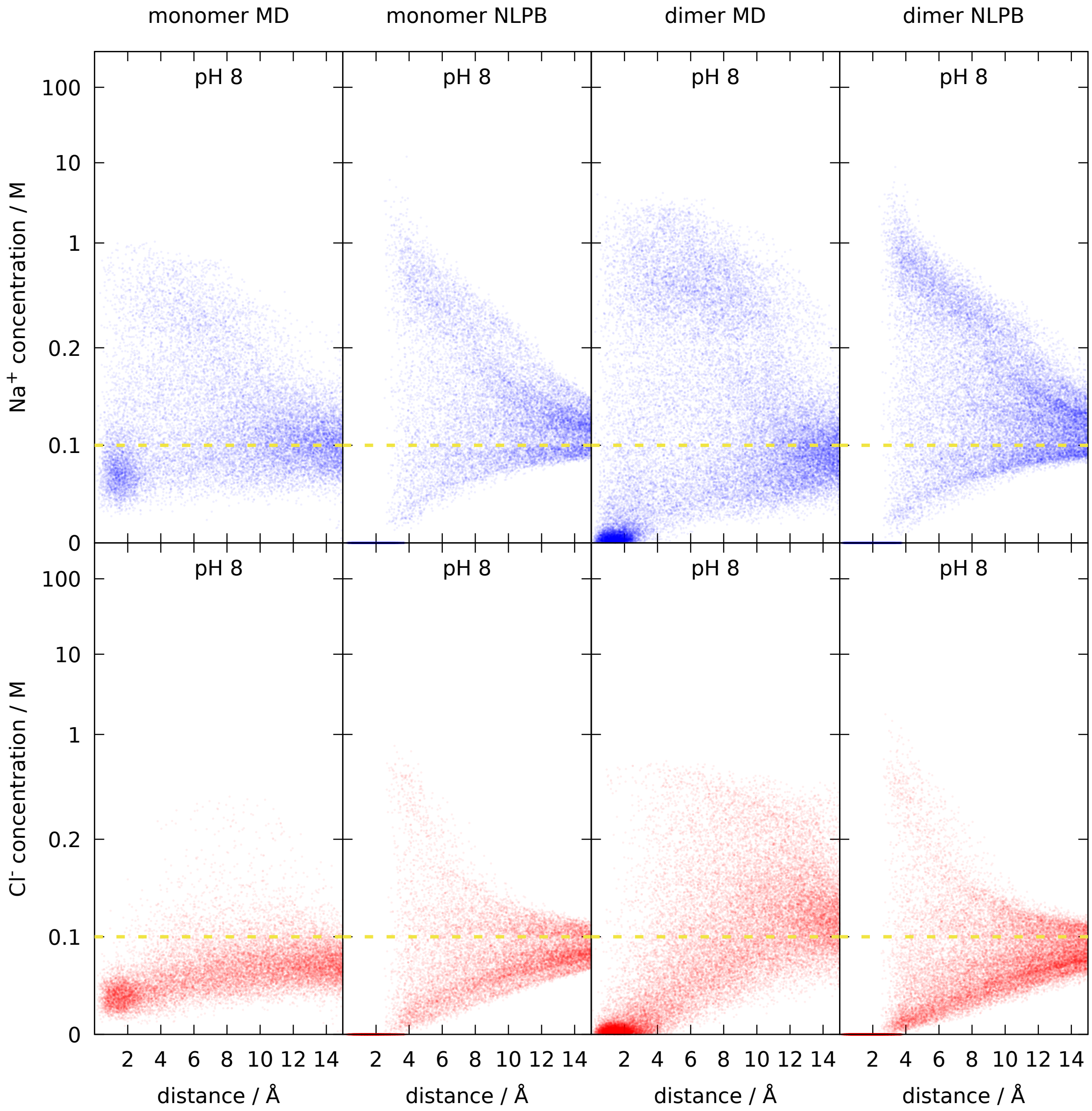

Figure S7: Scatter plots of Na^+ and Cl^- concentrations versus the distance from the closest protein atom at pH 8, found within a 15 Å distance from the protein. The scatter plots obtained by using the ion concentration maps from the MD or from the NLPB calculations are shown, for the monomer and dimer. The yellow dashed line indicates the bulk ionic strength of 0.1 M. The vertical scale is linear up to 0.2 M and logarithmic beyond that.

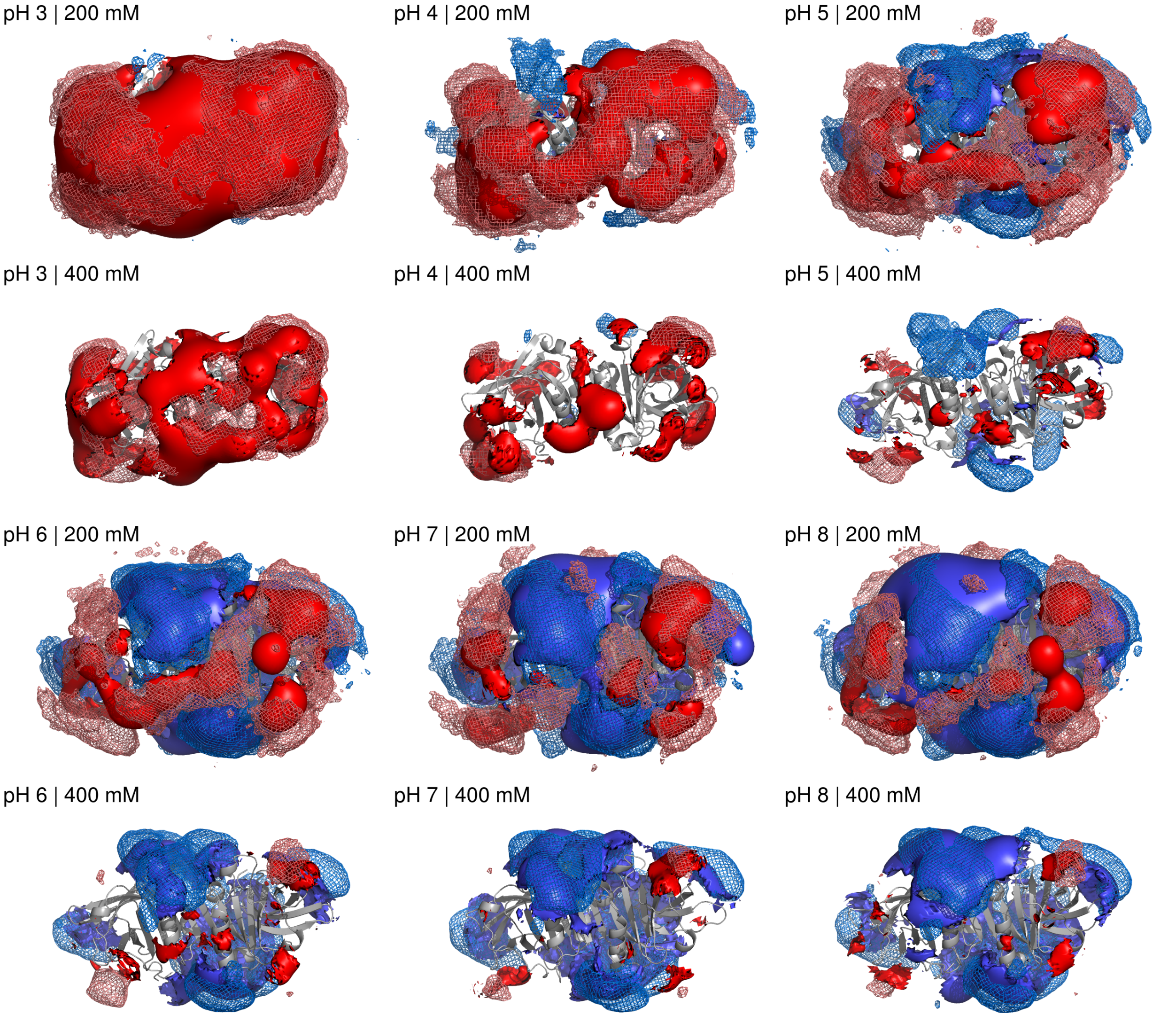

Figure S8: Ion iso-concentration contours of 200 mM (top) and of 400 mM (bottom) for Na^+ (blue) and Cl^- (red) in the dimer at different pH values, obtained from either the MD (mesh contours) or the NLPB (solid contours) concentration maps.

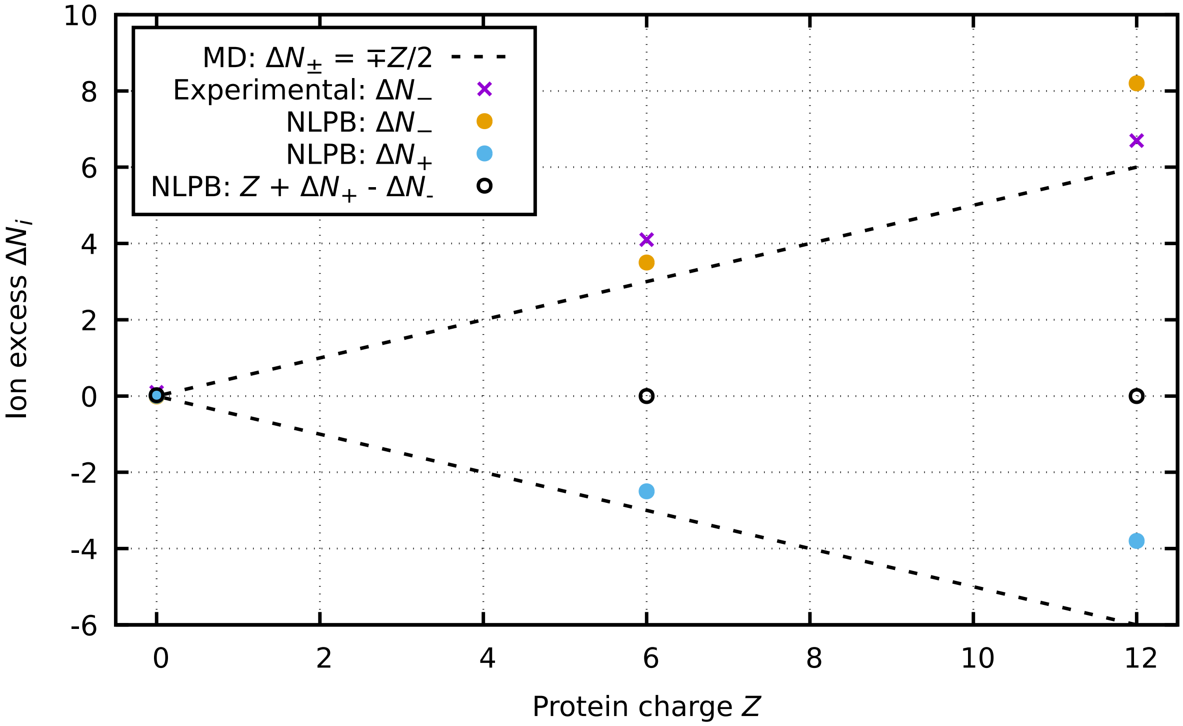

Figure S9: Experimental and NLPB-computed ion excess data versus protein charge Z, for Antp homeodomain ($Z = 12$), bovine pancreatic trypsin inhibitor ($Z = 6$) and ubiquitin ($Z = 0$). The plot shows anion excess ΔN_{-}, cation excess ΔN_{+}, and their sum with Z, taken from Yu et al. *Proc. Natl. Acad. Sci. U.S.A.* **2021**, 118, e2015879118. The dashed lines correspond to the protein half-charge condition used in our MD simulations.
